# Mental health problems of preschool children during the COVID-19 home quarantine: A cross-sectional study in Shanghai, China

**DOI:** 10.3389/fpsyg.2022.1032244

**Published:** 2022-11-01

**Authors:** Chen-huan Ma, Lian Jiang, Li-ting Chu, Chun-cao Zhang, Yuan Tian, Jin-jin Chen, Yu Wang

**Affiliations:** Department of Child Health Care, Shanghai Children’s Hospital, School of Medicine, Shanghai Jiao Tong University, Shanghai, China

**Keywords:** mental health, emotional and behavioral problems, preschool children, COVID-19, home quarantine

## Abstract

**Objective:**

As the coronavirus disease 2019 (COVID-19) pandemic spread across Shanghai, China, in late February 2022 and protective measures to mitigate its impact were enacted, this study aimed to estimate how home quarantine affected the mental health of preschool children in Shanghai, China and explore the association between lifestyle factors and mental health during this special period.

**Methods:**

A cross-sectional online survey of 2,110 preschool students from Shanghai, China, was conducted during May 20–25,2022. Preschooler’ mental health (Strengths and Difficulties Questionnaire, SDQ) and daily activities were reported by parents.

**Results:**

The sample involved 2,110 children with a mean age of 4.65 years [standard deviation (SD): 0.91, range: 3–6 years]. Boys and children whose mother’s education level were college and high school had higher rate of mental health problems. Boys had significantly higher rates of peer problems and prosocial behaviors than girls. The 3-year-old group had significantly higher rates of prosocial behaviors than other groups. As compared to the Shanghai norm and the SDQ results of preschool children in Shanghai in 2019 (SH2019), there were a significant decrease in emotional symptoms score, as well as a significant increase in conduct problems score. Additionally, peer problems score significantly increased compared to SH2019. Decreased time spent on daily sleep was associated with the increased risk for preschoolers’ mental health problems.

**Conclusion:**

There was an increase in the frequency of emotional and behavioral problems, especially regarding conduct problems and peer problems, in preschool children during the COVID-19 home quarantine in Shanghai, China. Boys, younger preschool children and children whose mother’s education level were college and high school may be especially vulnerable to emotional and behavioral problems. It was also found that decreased time spent on sleep may aggravate preschool children’s mental health problems. It may be beneficial to differentiate and focus on conducting psychoeducation and implementing psycho-behavioral interventions to solve these issues.

## Introduction

Coronavirus disease 2019 (COVID-19), caused by severe acute respiratory syndrome coronavirus 2 (SARS-CoV-2), has spread rapidly around the world. At present, the pandemic is still ongoing and even deteriorating in some regions and countries. A key measure to prevent COVID-19 infection in China is social distancing and isolation strategies, including home quarantine, working from home, and closing schools and public places. Although these measures were commendable and successful in preventing COVID-19 from spreading in China, concerns have been raised regarding the mental health of children during the COVID-19 pandemic (Liu et al., 2020; Wang et al., 2020; Ye, 2020). Despite being less susceptible to COVID-19 infection than adults and elders, children are not indifferent to the tremendous impact of the pandemic and even more likely to suffer adverse psychosocial effects. Children are aware of changes around them and are affected by them from as early as 2 years old (Imran et al., 2020). Since social distancing measures to reduce the COVID-19 spread have been required, there has been an abrupt withdrawal from school, social life, and outdoor activities among children, which has significantly disrupted their daily lives (de Figueiredo et al., 2021). When schools are closed for long periods of time and home quarantines are in place, children experience stress, such as frustration, boredom, lack of face-to-face contact with teachers and peers, and lack of private space at home. Concurrently, a combination of psychosocial stress and lifestyle changes could further exacerbate the negative effects on children’s mental health (Wang et al., 2020). Due to their heightened sensitivity to environmental risks, children are also less able to comprehend and deal with emergency situations on a short-and long-term basis (Crescentini et al., 2020). All the above-mentioned situations make children highly vulnerable during the COVID-19 home quarantine.

Previous research has shown that children during the pandemic-related quarantine were more likely to have mental health problems. Based on the findings of a preliminary study conducted in Shaanxi Province, China, clinginess, distraction, irritability, and fear of asking questions about the epidemic were the most common psychological and behavioral problems among children and adolescents (Jiao et al., 2020). Another study conducted in Shanghai, China, reported that anxiety, depression, and stress accounted for three of the most prevalent psychological symptoms among primary and secondary school students (Tang et al., 2021). Moreover, it has also been reported in India that a high prevalence of psychological distress was observed among children and adolescents quarantined, including helplessness, worry, and fear (Saurabh and Ranjan, 2020).

Although increasing attention has been paid to children’s mental health during this unusual period, attention to preschool children is still relatively inadequate compared with school-aged children. The preschool period is an essential period for the development of emotions and behavior, during which mental health is deeply ingrained. There is a high possibility that symptoms will develop in the same areas or overlap, and these problems may last a lifetime (Copeland et al., 2013). Childhood mental health problems also have considerable effects on children’s ability to earn and to work as adults as well as on intergenerational and intragenerational social mobility (Goodman et al., 2011). In addition, preschool children are more vulnerable to COVID-19 and exhibit more mental health problems than older children. Romero et al. (2020) reported that preschool children would be at increased risk of displaying more behavioral problems, including conduct problems and hyperactivity than older children. Schmidt et al. (2021) found that preschoolers had the largest increase in oppositional-defiant behaviors compared with school-aged children and adolescents. Furthermore, there is ample evidence showing that the reactions of differently aged children to COVID-19 vary significantly (Nearchou et al., 2020). Even during the preschool period, preschoolers of different ages exhibit different behavioral problems (Basten et al., 2016). As a result, it is essential to detect early signs of mental health problems among preschool children during the COVID-19 pandemic for preventing health problems later in life.

In addition to capturing preschool children’s mental health status during the COVID-19 pandemic, it also becomes especially important to explore how lifestyle factors influence preschooler’s mental health. According to the report by Jalal et al. (2021), COVID-19 confinement resulted in significantly reduced physical activity and increased sedentary time for children. The findings of a longitudinal study involving 14 countries reported that preschool children spent almost 1 h more per day on sedentary media, and went to bed 34 min later during the COVID-19 pandemic (Okely et al., 2021). However, empirical evidence regarding effects of changes in lifestyle on mental health of preschool children in China or other countries is still limited.

An outbreak of SARS-CoV-2 rapidly emerged in late February 2022 in Shanghai, China. During the spread of Omicron subvariant, the major cause of this outbreak, the city adopted a strategy of “citywide static management,” meaning a strict lockdown in which residents are prohibited from leaving their homes unless necessary. Preschool children in Shanghai have been at home for more than 2 months as of the time of investigation. Taking advantage of these gaps in the literature, we aimed to estimate the prevalence of emotional and behavioral problems among preschool children in Shanghai who experienced home quarantine and closure of schools. Further investigation was undertaken to determine the difference between the Shanghai norm and the SDQ results of preschoolers in Shanghai in 2019 (SH2019) in terms of emotional and behavioral problems. We also attempted to identify how changes in daily activities affect the mental health of preschool children. Having an understanding of these issues can help generate more robust evidence regarding COVID-19’s mental health effects on preschoolers that will help us gain a deeper understanding of how preschool children’s mental health has been affected during the COVID-19 home quarantine and develop better guidance for implementing more targeted mental health prevention/intervention programs.

## Materials and methods

### Participants

We followed the STROBE instructions for this study (see Supplementary material). This cross-sectional study was conducted among preschool children and their parents in central urban areas of Shanghai, China, on May 20–25, 2022, during a mandatory home quarantine. By using a cluster sampling method, Yangpu and Jing’an Distinct were randomly selected from seven districts in central urban areas of Shanghai. And six kindergartens were randomly sampled from Yangpu Distinct, and eight kindergartens were randomly sampled from Jing’an Distinct according to the proportion. All the preschoolers in the 14 identified kindergartens were recruited. Inclusion criteria were as follows: (1) participants were aged between 3 and 6 years; (2) participants were confined at home for 2 months at least; (3) the questionnaire should be able to be read, understood, and filled out independently by parents; (4) parents agreed to participant and provide online informed consent. Exclusion criteria was: parents refused to participant the survey.

### Measurements

#### Assessment of sociodemographic characteristics

Sociodemographic information included children’s age, gender (boys or girls), sibling (none, one, two or more), family type (extended family, nuclear family, or others), and parents’ educational level (master or above, college, high school, middle school or below).

#### Assessment of lifestyle factors

The effects of lifestyle factors (daily time spent on physical activity, media exposure and sleep) on preschool children’s mental health were examined. Daily time spent on physical activity before home quarantine and in the past month were reported separately and divided to equal or greater than 3 h, 2 h to lower than 3 h, 1 h to lower than 2 h, lower than 1 h. Daily media exposure before home quarantine and in the past month were reported separately and dichotomized to greater than 1 h versus lower or equal than 1 h. Daily time spent on sleep before home quarantine and in the past month were reported separately and divided to greater than 13 h, 10 h to lower than 13 h, lower than 10 h.

#### Assessment of children’s mental health

Mental health of preschool children was evaluated using the Strengths and Difficulties Questionnaire (SDQ; Goodman, 2007). Twenty-five items were asked to be rated by parents over the past 2 months, compositing five subscales: emotional symptoms, conduct problems, hyperactivity, peer problems, and prosocial behaviors. Three points were assigned to each item: 0 for not true, 1 for somewhat true, and 2 for definitely true. Throughout the subscales, a higher score indicated more problems, with the exception of the prosocial behavior subscale, where a lower score indicated more problems. For the purpose of determining whether overall support is necessary, the total difficulties score was calculated based on the scores for each subscale excluding “prosocial behaviors”. A higher score indicates a greater need for support. Du et al. (2008) introduced and adapted the SDQ to the Chinese language with accepted reliability and validity, and established Shanghai norm. For the Chinese version of parent-reported SDQ, the Cronbach’s alphas was 0.784, and the Pearson’s correlation coefficients was 0.717 over 6 weeks interval. It also had a good parallel validity with Parent Symptoms Questionnaire in behavioral and emotional problems of Chinese children and adolescents (Kou and Du, 2005). Cronbach’s alphas in the current sample was 0.783. In the Chinese version of parent-reported SDQ, cutoff scores were recommended to identify a child who may have challenges with emotional and behavioral problems (emotional symptoms score > 4, conduct problems score > 3, hyperactivity score > 7, peer problems score > 5, prosocial behaviors score < 5, and total difficulties score > 16; Du et al., 2008).

### Procedures

The survey was performed by Wenjuanxing,^1^ which is a frequently used online survey platform in China. Piloting the survey with several colleagues and getting their feedback on its content and functionality was done prior to distribution. The teachers of these kindergartens who had received training before the survey forwarded the survey link to Wechat groups in all classes, and the parents of each child were invited to fill out the questionnaire to report the performance of their child during this period. The parents’ consent was obtained before the survey. Prior to entering the study, participants were provided with consent information, including the purpose of the study, voluntariness of participation, confidentiality, duration, and data retention. The formal study was only accessible to parents who ticked the box “I understand information described above and agree to participate in this study”. Otherwise, the webpage window closed automatically. To ensure quality responses, the teachers of the kindergartens were instructed to provide help in answering questions. If parents have any questions about certain questions in the questionnaire, they can ask the teachers immediately; thereby, the questions can be clarified and explained. After completing the questionnaire, respondents will have the opportunity to review and change their answers. All the responses will be accessible by only the study investigator.

### Ethical considerations

Participants were informed about the study procedures and the ethical considerations, and informed consents were obtained online. The participation in this survey is entirely voluntary and anonymous. Participants’ and data confidentiality was ensured. Ethical approval was obtained prior to data collection from the Ethics Committee of Shanghai Children’s Hospital (approval number: 2021R060).

### Sample size

The sample size was determined with PASS 11. The total difficulties score of SDQ is primary outcome variable in this study. Alpha level was set to 0.05, confidence interval to 95%, and error level to 5%. As a result, a minimum sample of 1,537 participants were calculated. Based on the assumption that approximately 5% of the participants will be excluded from analysis, we targeted 1,690 participants for recruitment.

### Data collection

Among 2,135 preschool children, parents of nine individuals refused to participate, with a response rate of 99.6%. Among the respondents, 16 participants filled out the year of birth with 2022, which is an obvious mistake, and nine children did not match the age requirement. Their responses were excluded, resulting in a valid sample of 2,110 participants.

### Statistical analysis

The data of SDQ followed a normal distribution through a normality test. Statistical analysis was performed using IBM SPSS Statistics, version 24.0. Continuous variables were represented as mean (*M*) (standard deviation [SD]), while categorical variables were represented as frequency (percentage, %). A Chi-square test was utilized to compare the prevalence of emotional and behavioral problems among preschool children of different sociodemographic characteristics. Further analysis was conducted on children of various ages and genders with regard to the abnormal rates on SDQ subscales. A t-test was conducted to compare the difference in scores on emotional and behavioral problems between this study, Shanghai norm, and SH2019. Logistic regression analyses were used to evaluate the impact of lifestyle factors on mental health of preschool children, based on the crude model and the adjusted model. “The adjusted model was adjusted for children’s age, gender, sibling, family type and parents’ education level”. The odds ratios (OR), adjusted OR, and 95% confidence intervals (CI) were calculated. A *p* value of < 0.05 was considered statistically significant.

## Results

### Demographic characteristics

In this study, a total of 2,110 children aged 3–6 years (*M* = 4.65 years, SD = 0.91 years) were finally included. The participants comprised 1,076 boys (51.0%) and 1,034 girls (49.0%). Of the preschool children, 28.1% had at least one sibling. The proportion of the 3-year group, 4-year group, 5-year group, and 6-year group was 10.4, 34.2, 35.9 and 19.5%, respectively. Family type distribution was 63.5% with extended family, 34.6% with nuclear family and 1.9% with others. Father’s education level distribution was 11.6% with master or above, 77.7% with college, 8.8% with high school, and 1.9% with middle school or below. Mother’s education level distribution was 7.9% with master or above, 80.4% with college, 9.4% with high school, and 2.3% with middle school or below (Table 1).

**Table 1:** Prevalence of preschool children’s mental health problems by sociodemographic characteristics.

Variables	*N(%)*^a^	Strengths and Difficulties Questionnaire (SDQ)		*χ* ^2^	*p*
		Normal	Abnormal^b^		
Gender				7.171	0.007^*^
Boys	1,076 (51.0)	647 (48.8)	429 (54.8)		
Girls	1,034 (49.0)	680 (51.2)	354 (45.2)		
Age				4.220	0.239
3 years old	220 (10.4)	131 (9.9)	89 (11.4)		
4 years old	721 (34.2)	441 (33.2)	280 (35.8)		
5 years old	757 (35.9)	496 (37.4)	261 (33.3)		
6 years old	412 (19.5)	259 (19.5)	153 (19.5)		
Siblings				4.058	0.131
None	1,517 (71.9)	936 (70.5)	581 (74.2)		
One	559 (26.5)	371 (28.0)	188 (24.0)		
Two or more	34 (1.6)	20 (1.5)	14 (1.8)		
Family type				4.454	0.108
Extended family	1,340 (63.5)	833 (62.8)	507 (64.7)		
Nuclear family	730 (34.6)	474 (35.7)	256 (32.7)		
Others	40 (1.9)	20 (1.5)	20 (2.6)		
Father’s education level				6.261	0.100
Master or above	245 (11.6)	160 (12.1)	85 (10.9)		
College	1,639 (77.7)	1,041 (78.4)	598 (76.4)		
High school	185 (8.8)	105 (7.9)	80 (10.2)		
Middle school or below	41 (1.9)	21 (1.6)	20 (2.5)		
Mother’s education level				12.727	0.005^*^
Master or above	167 (7.9)	101 (7.6)	66 (8.4)^c^		
College	1,696 (80.4)	1,095 (82.5)	601 (76.8)		
High school	198 (9.4)	105 (7.9)	93 (11.9)		
Middle school or below	49 (2.3)	26 (2.0)	23 (2.9)^c^		

a The number and proportions of children within each category of sociodemographic characteristics.

b Emotional symptoms score > 4, conduct problems score > 3, hyperactivity score > 7, peer problems score > 5, prosocial behaviors score < 5, or total difficulties score > 16 indicates abnormal.

c Compared with children whose mother’s education level were college and high school, p < 0.01.

* *p* < 0.01.

### Prevalence of mental health problems

Table 1 shows the percentages of preschool children’s mental health problems by sociodemographic characteristics. Any SDQ subscale that met the cutoff score was thought that the child was suffering from mental health problems. Among the participants, 783 children (37.1%) demonstrated mental health problems. Compared with girls, boys were more likely to have mental health problems (*χ*^2^ = 7.171, *p* < 0.01). The prevalence of mental health problems was higher among those whose mother’s education level were college and high school (*χ*^2^ = 12.727, *p* < 0.01).

Table 2 provides the percentages that met cutoff scores for emotional symptoms, conduct problems, hyperactivity, peer problems, prosocial behaviors, and total difficulties by gender and age. Among the participants, 24.98% experienced peer problems, followed by 12.89% who experienced conduct problems and 9.05% who experienced hyperactivity. Boys had significantly higher rates of peer problems and prosocial behaviors than girls (*χ*^2^ = 18.856, 19.663, *p* < 0.01). The 3-year-old group had significantly higher rates of prosocial behaviors than other groups (*χ*^2^ = 13.399, *p* < 0.05).

**Table 2:** Comparison of detection rates of emotional and behavioral problems in preschool children by gender and age.

Items	Boys (*n* = 1,076)	Girls (*n* = 1,034)	*χ* ^2^	*p*	3-year age group (*n* = 220)	4-year age group (*n* = 721)	5-year age group (*n* = 757)	6-year age group (*n* = 412)	*χ* ^2^	*p*
Emotional Symptoms	30(2.8%)	41(4.0%)	2.927	0.231	6(2.7%)	21(2.9%)	30(4.0%)	14(3.4%)	2.785	0.835
Conduct Problems	134(12.5%)	138(13.3%)	0.671	0.751	28(12.7%)	100(13.9%)	93(12.3%)	51(12.4%)	7.362	0.289
Hyperactivity	95(8.8%)	96(9.3%)	5.014	0.081	23(10.5%)	67(9.3%)	68(9.0%)	33(8.0%)	7.317	0.293
Peer Problems	304(28.3%)	223(21.6%)	18.856	0.000^**^	54(24.5%)	189(26.2%)	176(23.2%)	108(26.2%)	9.320	0.156
Prosocial Behavior	61(5.7%)	30(2.9%)	19.663	0.000^**^	18(8.2%)	34(4.7%)^a^	24(3.2%)^a^	15(3.6%)^a^	13.399	0.037^*^
Total Difficulties	89(8.3%)	90(8.7%)	2.805	0.246	19(8.6%)	61(8.5%)	63(8.3%)	36(8.7%)	1.427	0.964

* *p* < 0.05 and

** *p* < 0.01.

a Compared with 3-year age group, *p* < 0.05.

### Comparison with Shanghai norm

A comparison of emotional and behavioral problems scores between this study and Shanghai norm is shown in Table 3. This study found that emotional symptoms score of 4–5-year-old boys (*t* = −3.026, *p* < 0.01; *t* = −2.084, *p* < 0.05) and 4–6-year-old girls (*t* = −2.126, *p* < 0.05; *t* = −3.040, *p* < 0.01; *t* = −4.331, *p* < 0.01) was significantly lower than that in Shanghai norm. Conduct problems score of 5–6-year-old boys (*t* = 2.574, 2.429, *p* < 0.05) and 4–6-year-old girls (*t* = 6.392, 3.124, 3.542, *p* < 0.01) was significantly higher than that in the Shanghai norm. Six-year-old children in this study showed a significantly lower level of hyperactivity score than Shanghai norm (*t* = −2.586, −2.214, *p* < 0.05). There was a significantly higher score in peer problems of 4–5-year-old boys compared with Shanghai norm (*t* = 2.961, *p* < 0.01; *t* = 2.224, *p* < 0.05). Girls aged 4 years scored significantly higher in total difficulty score compared to the Shanghai norm (*t* = 2.139, *p* < 0.05).

**Table 3:** Comparison of preschool children’s SDQ scores with the Shanghai norm.

Age		4-year age group		*t*	*p*	5-year age group		*t*	*p*	6-year age group		*t*	*p*
Group		This study	The Shanghai norm			This study	The Shanghai norm			This study	The Shanghai norm		
Total		721	189			757	180			412	155		
Emotional Symptoms	Boys	1.45(1.33)	1.94(1.41)	−3.026	0.003^**^	1.50(1.43)	1.85(1.60)	−2.084	0.038^*^	1.53(1.37)	1.78(1.75)	−1.166	0.246
	Girls	1.57(1.45)	1.96(1.70)	−2.126	0.035^*^	1.54(1.41)	2.12(1.62)	−3.040	0.003^**^	1.49(1.35)	2.68(2.20)	−4.331	0.000^**^
Conduct Problems	Boys	1.98(1.38)	1.71(1.20)	1.811	0.072	1.89(1.35)	1.50(1.23)	2.574	0.010^*^	1.88(1.36)	1.46(1.28)	2.429	0.016^*^
	Girls	2.04(1.39)	1.25(1.02)	6.392	0.000^**^	1.80(1.33)	1.36(1.13)	3.124	0.002^**^	1.83(1.42)	1.23(1.14)	3.542	0.001^**^
Hyperactivity	Boys	4.05(1.82)	4.45(2.31)	−1.495	0.138	4.06(2.04)	4.53(2.30)	−1.961	0.050	3.99(1.96)	4.73(2.30)	−2.586	0.011^*^
	Girls	3.94(2.02)	3.63(2.24)	1.270	0.206	3.70(1.91)	4.01(2.28)	−1.161	0.248	3.53(1.99)	4.16(2.23)	−2.214	0.028^*^
Peer Problems	Boys	2.66(1.56)	2.10(1.60)	2.961	0.003^**^	2.63(1.49)	2.25(1.52)	2.224	0.027^*^	2.45(1.61)	2.52(1.50)	−0.342	0.733
	Girls	2.39(1.53)	2.09(1.59)	1.749	0.081	2.17(1.49)	2.21(1.37)	−0.226	0.821	2.44(1.53)	2.36(1.53)	0.380	0.704
Prosocial Behavior	Boys	7.07(1.94)	6.98(1.79)	0.390	0.696	7.43(1.89)	7.20(1.90)	1.063	0.288	7.44(1.90)	7.26(1.64)	0.814	0.417
	Girls	7.67(1.83)	7.39(1.78)	1.383	0.167	7.92(1.77)	7.83(1.71)	0.425	0.671	7.78(1.83)	7.59(1.87)	0.748	0.455
Total Difficulties	Boys	10.14(4.20)	10.20(4.25)	−1.118	0.906	10.08(4.65)	10.19(4.10)	−0.212	0.833	9.84(4.34)	10.48(4.25)	−1.149	0.251
	Girls	9.95(4.72)	8.93(4.16)	2.139	0.034^*^	9.20(4.53)	9.70(3.91)	−0.939	0.348	9.29(4.67)	10.43(4.83)	−1.752	0.081

* *p* < 0.05 and

** *p* < 0.01.

SDQ, strengths and difficulties questionnaire; Data are expressed as the means (SD).

### Comparison with SH2019

Table 4 presents the comparison of emotional and behavioral problems scores between this study and SH 2019. As compared to SH2019, boys aged 4–5 years (*t* = −4.415, −4.300, *p* < 0.01) and girls aged 4–6 years (*t* = −3.819, −5.678, −2.875, *p* < 0.01) had significantly lower emotional symptom score in this study. Both boys and girls aged 4 years (*t* = 2.750, 5.520, *p* < 0.01) and 6 years (*t* = 2.613, *p* < 0.05; *t* = 2.976, *p* < 0.01) had significantly higher conduct problems score compared to SH2019. There were significantly higher hyperactivity score among 4-year-old girls (*t* = 3.280, *p* < 0.01) and lower hyperactivity score among 5-year-old children (*t* = −3.301, *p* < 0.01; *t* = −2.205, *p* < 0.05) compared to SH2019. Among children aged 4–6 years, peer problem scores were significantly higher in this study than in SH2019 (*t* = 6.112, 5.696, 6.362, 4.768, 4.714, and 6.576, *p* < 0.01). In this study, children aged 5 years scored significantly higher for prosocial behavior (*t* = 3.249, *p* < 0.01; *t* = 2.403, *p* < 0.05), and children aged 4 years scored significantly higher for total difficulties score compared to SH2019 (*t* = 2.409, *p* < 0.05, *t* = 3.735, *p* < 0.01).

**Table 4:** Comparison of preschool children’s SDQ scores with SH2019.

Age		4-year age group		*t*	*p*	5-year age group		*t*	*p*	6-year age group		*t*	*p*
Group		This study	SH2019			This study	SH2019			This study	SH2019		
Total		721	9,178			757	7,132			412	7,015		
Emotional Symptoms	Boys	1.45(1.33)	1.78(1.54)	−4.415	0.000^**^	1.50(1.43)	1.84(1.67)	−4.300	0.000^**^	1.53(1.37)	1.66(1.63)	−1.382	0.168
	Girls	1.57(1.45)	1.87(1.60)	−3.819	0.000^**^	1.54(1.41)	1.98(1.68)	−5.678	0.000^**^	1.49(1.35)	1.79(1.63)	−2.875	0.004^**^
Conduct Problems	Boys	1.98(1.38)	1.77(1.29)	2.750	0.006^**^	1.89(1.35)	1.85(1.32)	0.557	0.578	1.88(1.36)	1.64(1.27)	2.613	0.010^*^
	Girls	2.04(1.39)	1.63(1.23)	5.520	0.000^**^	1.80(1.33)	1.69(1.25)	1.624	0.104	1.83(1.42)	1.51(1.19)	2.976	0.003^**^
Hyperactivity	Boys	4.05(1.82)	3.92(2.41)	1.251	0.212	4.06(2.04)	4.43(2.30)	−3.301	0.001^**^	3.99(1.96)	4.15(2.30)	−1.192	0.234
	Girls	3.94(2.02)	3.58(2.25)	3.280	0.001^**^	3.70(1.91)	3.93(2.21)	−2.205	0.028^*^	3.53(1.99)	3.75(2.11)	−1.370	0.171
Peer Problems	Boys	2.66(1.56)	2.11(1.62)	6.112	0.000^**^	2.63(1.49)	2.09(1.57)	6.362	0.000^**^	2.45(1.61)	1.95(1.56)	4.714	0.000^**^
	Girls	2.39(1.53)	1.91(1.57)	5.696	0.000^**^	2.17(1.49)	1.79(1.48)	4.768	0.000^**^	2.44(1.53)	1.72(1.43)	6.576	0.000^**^
Prosocial Behavior	Boys	7.07(1.94)	7.08(2.03)	0.089	0.929	7.43(1.89)	7.09(1.93)	3.249	0.001^**^	7.44(1.90)	7.52(1.86)	−0.633	0.527
	Girls	7.67(1.83)	7.60(1.91)	0.684	0.494	7.92(1.77)	7.69(1.78)	2.403	0.016^*^	7.78(1.83)	7.97(1.74)	−1.427	0.154
Total Difficulties	Boys	10.14(4.20)	9.57(4.87)	2.409	0.016^*^	10.08(4.65)	10.20(4.72)	−0.469	0.639	9.84(4.34)	9.39(4.74)	1.521	0.129
	Girls	9.95(4.72)	9.00(4.73)	3.735	0.000^**^	9.20(4.53)	9.39(4.60)	−0.769	0.442	9.29(4.67)	8.n(4.46)	1.495	0.135

* *p* < 0.05 and

** *p* < 0.01.

SDQ, strengths and difficulties questionnaire; SH2019, the SDQ results of preschool children in Shanghai in 2019; Data are expressed as the means (SD).

### Lifestyle factors associated with mental health problems

Table 5 presented the risks of preschoolers’ mental health associated with lifestyle factors. Increased time spent on daily physical activity (OR = 0.72, 95% CI, 0.53–0.97, *p* < 0.05) and decreased time spent on daily media exposure (OR = 0.52; 95% CI, 0.29–0.95, *p* < 0.05) were associated with the decreased risk for preschoolers’ mental health problems. Decreased time spent on daily sleep (OR = 1.63; 95% CI, 1.08–2.45, *p* < 0.05) was associated with the increased risk for preschoolers’ mental health problems. After controlling for sociodemographic characteristics, preschoolers who had decreased time spent on daily sleep (OR = 1.63; 95% CI, 1.70–2.50, *p* < 0.05) were more likely to develop mental health problems.

**Table 5:** Associations of preschool children’s mental health problems with lifestyle factors.

	ORs (95% CI) for risks of mental health problems^a^			
	Unadjusted OR (95% CI)	*p*	Adjusted OR^b^ (95% CI)	*p*
Time spent on daily physical activity				
Decreased	1.00		1.00	
No change	1.07 (0.88, 1.29)	0.499	1.02 (0.84, 1.26)	0.820
Increased	0.72 (0.53, 0.97)	0.032^*^	0.74 (0.54, 1.01)	0.057
Time spent on daily media exposure				
Increased	1.00		1.00	
No change	0.62 (0.34, 1.12)	0.111	0.65 (0.35, 1.20)	0.167
Decreased	0.52 (0.29, 0.95)	0.033^*^	0.57 (0.31, 1.07)	0.080
Time spent on daily sleep				
Increased	1.00		1.00	
No change	0.96 (0.75, 1.24)	0.764	0.95 (0.72, 1.24)	0.686
Decreased	1.63 (1.08, 2.45)	0.019^*^	1.63 (1.07, 2.50)	0.024^*^

a Emotional symptoms score > 4, conduct problems score > 3, hyperactivity score > 7, peer problems score > 5, prosocial behaviors score < 5, or total difficulties score > 16 indicates mental health problems.

b Adjusted by children’s age, gender, sibling, family type and parents’ education level.

* *p* < 0.05.

## Discussion

This Internet-based cross-sectional study assessed the mental health of preschool children by SDQ during home quarantine due to COVID-19 in Shanghai, China. Based on this study, the prevalence of emotional and behavioral problems was higher than that in Minhang Distinct of Shanghai (6.6%; Deng et al., 2019), Guangming Distinct of Shenzhen (6.5%; Zhang et al., 2020), and Beijing (6.01%; Cui and Wang, 2017) prior to the COVID-19 pandemic. However, the prevalence observed in this study was lower than that reported in previous studies during the COVID-19 period. In May 2021, Wang et al. (2021) reported that 11.2–35.2% of 16,094 preschool children from 28 provinces across mainland China had emotional and behavioral problems. Another study, which included 504 preschool children from mainland China in April 2021, reported that 10.3–25.8% of preschoolers suffered from emotional and behavioral problems (Wang et al., 2021). The major reason for the difference is that the studies have been conducted in different regions of mainland China between different periods. We also speculate that the importance attached to the mental health of children by the government, organizations, hospitals, and schools during the COVID-19 period could be a contributing factor. International organizations and advisory bodies have released various guidelines aimed at universal prevention and mental health promotion during the COVID-19 period. For example, a tool called “Helping Children Cope with Stress During the 2019-CoV Outbreak” has been developed by the World Health Organization (WHO, 2020). Parents are advised to discuss the current pandemic with their children in accordance with children’s maturity level and ability to comprehend the crisis. When children do not know about what is happening around, they tend to worry more and often express worry in the form of anger, distraction, and temper tantrums (Panda et al., 2021). As a result, open and frequent communication between parents and children can serve as protective factors for their mental health. Additionally, it is significant for children to keep regular routines and schedules as much as possible during home quarantine. Similarly, many organizations have initiated specialist mental health services to support children with mental health problems at this challenging time, including text-messaging and telephone services (Meherali et al., 2021). Psychologists, psychiatrists, pediatricians, and community health practitioners also have taken action without violating the lockdown and social distancing norms to reach the children at every corner of the community.

Boys had significantly more problems than girls regarding peer problems and prosocial behaviors. Such gender differences were also found in previous studies. Gustafsson et al. (2017) found that boys had significantly more problems than girls on the entire scale of SDQ, and on all subscales except for the emotional subscale. Wang et al. (2021) reported that boys were more likely to have abnormal prosocial behavior scores compared with the counterparts. Due to gender-related differences in gene expression and hormonal levels, boys and girls perform significantly differently on psychological and behavioral measures, which might account for the differences in the incidence of mental health problems (Ford et al., 2003). The current results also revealed that three-year-old children were considered to have significantly less prosocial skills than children of other age groups. Socialization begins for preschool children at the age of 3 years when they enter preschool from home. And social networks expand gradually as children grow up. Unfortunately, although three-year-old children have strong motivation to communicate with other individuals, long-term home isolation inhibits their peer communication, further supporting their socialization. It has been recommended that more time and attention should be devoted on younger children compared with adolescents, during the COVID-19 pandemic and lockdown (Singh et al., 2020). At the same time, it is also crucial to attach more significance on younger preschoolers.

The differences between this study, the Shanghai norm, and SH2019 comprise a significant decrease in emotional symptoms score and a significant increase in conduct problems score. Additionally, peer problems score significantly increased compared to SH2019. Parental involvement can provide an important context for children’s socioemotional development, especially during times of extreme stress and uncertainty (Barger et al., 2019). To counter the spread of COVID-19, China has implemented a number of measures, including home quarantines, home working, and school closure, resulting in parents having unprecedented amounts of time at home with their children. Parents’ higher involvement has been associated with children’s lower levels of emotional distress, including anxiety/withdrawal, fearfulness, and acting out (Zhang, 2021). Therefore, parents’ involvement may be a key to reducing preschool children’s emotional distress during the COVID-19 quarantine. However, a study by Zhang (2021) found that primary caregivers of preschool children spent less time on their children’s education at home during the pandemic than they were before. Because pandemic-related emotional symptoms may not be immediately apparent, they will likely be detected after a period of time, and we need to identify these symptoms as soon as possible. Compared to the general population, children is the group who may be more sensitive to routine changes (Wang et al., 2020). Significant changes in daily routine that children have experienced during home quarantine which are vital factors for healthy development might interfere with their sense of predictability and security (Cusinato et al., 2020). As humans, we cannot tolerate spatial distancing in normal circumstances. This is more pronounced during the COVID-19 pandemic when affiliative tendencies and the desire to seek physical contact become even stronger (Tomasello, 2014). The above-mentioned reasons, with the addition of anxiety, fear, and feeling of unpredictability, possibly contribute to the significant increase in conduct problems and peer problems scores. Additionally, as a group who are also equally stressed out, parents, especially mothers, can contribute to the worsening of mental health of children (Yeasmin et al., 2020). A few studies have confirmed that an increase in children’s emotional and behavioral problems were in connection with parents’ depression and anxiety symptoms during the pandemic (Tokunaga et al., 2019; Patrick et al., 2020; Kim et al., 2021). The rise in parents’ anxiety during the COVID-19 outbreak may stem from the health, economic, and social emergencies related to the pandemic, and its accompanying containment measures might not only have occupied caregivers’ time but also caused their stress (Spinelli et al., 2020). The measures taken to control the virus spread have leaded to parents pursuing the balance between working at home and regulating their children’s daily routines. As a result, this sudden overload has been putting parents under extra stressful conditions, potentially increasing the risk of children facing emotional and behavioral problems (Dollberg et al., 2021). Additionally, parental stress has been linked to higher levels of family conflict, and, through parental behavior, it contributes to the creation of a chaotic family environment, which, in turn, puts the child at risk for behavioral problems (Cusinato et al., 2020).

The findings in this study suggest important implications for public health. In the COVID-19 epidemic home quarantine, more attention should be given to preschool children’s mental health, particularly boys, younger preschool children and preschoolers whose mother’s education level were college and high school. One potential way to reduce the risk on mental health would to be getting enough sleep, since sleep deprivation can increase the possibility of developing mental health problems for preschool children. It is necessary to deliver mental health prevention or intervention programs for preschool children who are undergoing during the COVID-19 home quarantine in a more targeted manner.

## Limitations and future directions

In the present study, we evaluated the emotional and behavioral impact of home quarantine on preschool children in Shanghai, China, during the COVID-19 pandemic and discovered lifestyle factors influencing children’s mental health. There were some limitations to the study. First, the participants only covered the preschool children from Jing’an district and Yangpu district, which could lead to some bias in the results of the study. Additionally, we did not collect information about the baseline emotional and behavioral problems of the same group of preschool children before the COVID-19 outbreak. Although we used historical controls to analyze changes in emotional and behavioral problems among preschool children, differences in demographic characteristics could have an impact on the results of the study. Finally, the mental health ramifications of COVID-19 home quarantine are likely to be longstanding but not simply chronic. New difficulties for children who initially appeared well-adapted might surface later in their development (Wade et al., 2020). It is recommended to conduct a long-term cohort study for long-term follow-up.

## Conclusion

The current study showed that the prevalence of emotional and behavioral problems, especially regarding conduct problems and peer problems, is higher than before in preschool children during the COVID-19 epidemic home quarantine in Shanghai, China. Boys, younger preschool children and children whose mother’s education level were college and high may be especially vulnerable to emotional and behavioral problems. This study also found that decreased time spent on sleep may aggravate preschool children’s mental health problems. Therefore, it is necessary to attach great importance of preschoolers’ mental health during the COVID-19 home quarantine and deliver mental health prevention or intervention programs in a more targeted and distinctive manner.

## Data availability statement

The raw data supporting the conclusions of this article will be made available by the authors, without undue reservation.

## Ethics statement

The studies involving human participants were reviewed and approved by Ethics Committee of Shanghai Children's Hospital. Written informed consent to participate in this study was provided by the participants' legal guardian/next of kin.

## Author contributions

YW, J-jC, and C-hM designed the study. LJ and YT collected the data. L-tC and C-cZ performed the statistical analysis. C-hM and LJ completed the manuscript. All authors contributed to the article and approved the submitted version.

## Funding

This work was supported by Shanghai Municipal Health Commission (grant number: GWV-10.1-XK 19, 202140299).

## Conflict of interest

The authors declare that the research was conducted in the absence of any commercial or financial relationships that could be construed as a potential conflict of interest.

## Publisher’s note

All claims expressed in this article are solely those of the authors and do not necessarily represent those of their affiliated organizations, or those of the publisher, the editors and the reviewers. Any product that may be evaluated in this article, or claim that may be made by its manufacturer, is not guaranteed or endorsed by the publisher.

## Footnotes

^1^ www.wjx.cn
